# Using mathematical modeling to inform health policy: A case study from voluntary medical male circumcision scale-up in eastern and southern Africa and proposed framework for success

**DOI:** 10.1371/journal.pone.0213605

**Published:** 2019-03-18

**Authors:** Emmanuel Njeuhmeli, Melissa Schnure, Andrea Vazzano, Elizabeth Gold, Peter Stegman, Katharine Kripke, Michel Tchuenche, Lori Bollinger, Steven Forsythe, Catherine Hankins

**Affiliations:** 1 United States Agency for International Development, Washington, District of Columbia, United States of America; 2 Project SOAR (Supporting Operational AIDS Research), Palladium, Washington, District of Columbia, United States of America; 3 AIDSFree, JSI Research and Training Institute, Arlington, Virginia, United States of America; 4 Project SOAR (Supporting Operational AIDS Research), Avenir Health, Washington, District of Columbia, United States of America; 5 Department of Global Health and Amsterdam Institute for Global Health and Development, University of Amsterdam, Amsterdam, the Netherlands; 6 Department of Infectious Disease Epidemiology, Faculty of Epidemiology and Population Health, London School of Hygiene and Tropical Medicine, London, United Kingdom; Burnet Institute, AUSTRALIA

## Abstract

**Background:**

Modeling contributes to health program planning by allowing users to estimate future outcomes that are otherwise difficult to evaluate. However, modeling results are often not easily translated into practical policies. This paper examines the barriers and enabling factors that can allow models to better inform health decision-making.

**Description:**

The Decision Makers’ Program Planning Tool (DMPPT) and its successor, DMPPT 2, are illustrative examples of modeling tools that have been used to inform health policy. Their use underpinned Voluntary Medical Male Circumcision (VMMC) scale-up for HIV prevention in southern and eastern Africa. Both examine the impact and cost-effectiveness of VMMC scale-up, with DMPPT used initially in global advocacy and DMPPT 2 then providing VMMC coverage estimates by client age and subnational region for use in country-specific program planning. Their application involved three essential steps: identifying and engaging a wide array of stakeholders from the outset, reaching consensus on key assumptions and analysis plans, and convening data validation meetings with critical stakeholders. The subsequent DMPPT 2 Online is a user-friendly tool for in-country modeling analyses and continuous program planning and monitoring.

**Lessons learned:**

Through three iterations of the DMPPT applied to VMMC, a comprehensive framework with six steps was identified: (1) identify a champion, (2) engage stakeholders early and often, (3) encourage consensus, (4) customize analyses, (5), build capacity, and (6) establish a plan for sustainability. This framework could be successfully adapted to other HIV prevention programs to translate modeling results to policy and programming.

**Conclusions:**

Models can be used to mobilize support, strategically plan, and monitor key programmatic elements, but they can also help inform policy environments in which programs are conceptualized and implemented to achieve results. The ways in which modeling has informed VMMC programs and policy may be applicable to an array of other health interventions.

## Introduction

### Introduction to mathematical modeling

In the health sphere, mathematical modeling is a key tool for decision-making, particularly when direct evaluation of interventions is not an option. Mathematical modeling is governed by a set of equations or rules describing a real-life dynamic system and is dependent upon key assumptions about the relationship between input parameters and outcomes [1]. While modern mathematical modeling for public health purposes has become more complex and refined over time, we can trace its origins far back in history. In the 1700s, Swiss mathematician Daniel Bernoulli used smallpox mortality projections to make the case for increased vaccination, even without a complete understanding of all influencing factors [2].

Modeling is often the only viable and timely option for informing quick decision-making when direct experimentation is time consuming, unethical, or impractical, especially when projecting into the future. Randomized controlled trials, considered the ‘gold standard’ for informing clinical decisions, are not always feasible. Population-based surveys are useful, but they provide only point estimates in the year in which data are collected. Such surveys, which cannot project into the future, often are not powered to sub-national levels or disaggregated appropriately by age, limiting their utility. In contrast, modeling can account for changing demographics over time, such as aging, as well as vital dynamics such as mortality, and births.

Policy and health decision-makers are often encouraged to make greater use of a range of tools and systems in supporting their staff when formulating and implementing health policies [3]. Translation of modeling knowledge is key for effective communication between modelers, policy-makers and implementers [4]. Although mathematical modeling has informed health policy decisions in various health- related matters, research utilization of real-world impact models could be low [3]. Thus, documenting successful use of a comprehensive framework for knowledge translation of modelling to policy and programming is important.

### Barriers and enablers to evidence use

The suboptimal use of research to inform health policy is due to numerous barriers [1]. Given the demonstrable utility of modeling for program planning, it is crucial to consider why modeling results may not inform policy environments. Historically, a disconnect between modelers and policymakers often has made it difficult to translate model results into practical policies. In exploring the literature on this issue, we identified several barriers and enablers to the use of modelled evidence. Some of the following examples deal explicitly with modeling results, while others discuss general research evidence that is in turn utilized by models.

#### Barriers

One barrier to the use of evidence arises during the conceptualization of a modeling or research study: *failure to consider the end-user*. There are several examples of this in the literature. In a discussion of diffusion theory and knowledge dissemination, Green et al. point to “tradition-bound practitioners” and “smug scientists” who believe that “if they publish it, practitioners and the public will use it” [5]. In a study of the promotion of evidence-informed policymaking, one barrier to evidence use was undertaking research that was not relevant for decision-making [6]. A qualitative study of how research evidence influences public health policymaking describes how a key bottleneck occurs when researchers “take little account of the needs of policymakers” [7]. Each of these descriptions point to one common barrier to knowledge translation: researchers and modelers conducting studies for their own purposes and not considering from the outset the potential use in the “real world”.

The second barrier occurs at the point of absorption of research evidence: a *lack of understanding on the users’ end*. In discussing missed opportunities for using models in decision-making in the United States healthcare system, Mandelblatt et al. found that models were often perceived as “black boxes” whose inner workings are a mystery to the end-user [8]. Lacking knowledge of what goes into a model, what assumptions are made, and the relative significance of the results, policymakers may find modeling results daunting to understand [1].

#### Enablers

Our literature review identified several enablers of evidence use. First, there is overwhelming agreement that *collaboration with policymakers—particularly early*, *continued collaboration*—facilitates eventual translation of evidence into policy. The qualitative study mentioned above highlights that “the earlier you bring policy-makers into the evaluation process, the better the outcome” [9]. This suggestion is echoed in several other studies that highlight early collaboration as central to research utilization by stakeholders and policymakers [6,10–13].

A second enabler focuses on context: rather than ignore the end-user, successful studies give due *consideration to the local context in which results will eventually be applied*. In examining the gap between research and practice, Glasgow et al. conclude that there needs to be a “greater understanding of, and research on, setting-level social contextual factors” [14] that influence knowledge translation. For evidence to eventually transform into policy, researchers and modelers must consider the local, practical implications of their findings [7, 12].

The third enabler is key to removing the “black box” effect discussed earlier: *up-front acknowledgment of assumptions and limitations*. A report on the use of mathematical modeling in developing the 2013 World Health Organization (WHO) antiretroviral therapy (ART) guidelines concludes that an important consideration to guide future modeling use is “transparency in the conduct and reporting of model inputs and results” [13]. By fully disclosing model parameters and assumptions—and effectively communicating these assumptions—modelers can ensure their results are more accessible to policymakers who may lack a strong understanding of modelling while policymakers and others can ensure that parameter inputs into models more accurately reflect local environments.

The fourth enabler identified is: *sufficient commitment of time to a thorough analysis*. Modeling analyses should not be an afterthought, but rather incorporated into the larger conversation. One review of modeling approaches concludes that consideration must be given to “… the time and resources needed for good quality modeling as part of evaluation, rather than modeling being an inconvenient and rushed add-on” [1].

To inform formulation of comprehensive, practical guidance, we discuss a successful case study of modeling for health policy in the following section.

## Case study: Voluntary medical male circumcision

### Background

In the mid-2000s, three randomised controlled trials [15–17] demonstrated that VMMC provides partial protection for men against HIV acquisition of approximately 60%. Considering these results and evidence that VMMC offers several other health benefits [18–21], the World Health Organization (WHO) and the Joint United Nations Programme on HIV/AIDS (UNAIDS) identified VMMC as a priority HIV prevention intervention in 2007 in areas with high HIV prevalence and low levels of male circumcision [22]. The 13 countries prioritized in eastern and southern Africa were Botswana, Kenya, Lesotho, Malawi, Mozambique, Namibia, Rwanda, South Africa, Swaziland, Tanzania, Uganda, Zambia, and Zimbabwe [22]. An ambitious goal was set to rapidly scale-up male circumcision coverage to 80% in these 13 countries. It was recognized that an intense global advocacy effort was needed to mobilize the considerable technical and financial resources needed from country governments and international donors to achieve this goal. Modeling was used to inform global policymakers about both the costs and the benefits of scaling up VMMC.

As countries developed and implemented national VMMC programs, modelling was again used to support revisions and clarifications of initial VMMC coverage targets and explore answers to questions about what impacts could be expected if specific age groups or geographical areas were prioritized. More recently, countries have been using VMMC modeling to track program progress down to various sub-national levels. The following three sections highlight the different iterations of modelling for VMMC and detail how each phase was uniquely tailored to a new and more complex policy environment.

### The Decision Makers’ Program Planning Tool (DMPPT): VMMC modeling to support global advocacy

In 2008, after the randomized controlled trials for VMMC were published, UNAIDS requested modeler John Stover and Economist Lori Bollinger at Futures Institute (now Avenir Health) to estimate the epidemiologic impact and cost-effectiveness associated with various VMMC scale-up scenarios in high HIV prevalence settings [23–25]. Stover and Bollinger then developed the maiden version of the Decision Makers’ Program Planning Tool (DMPPT). The subsequent modeling exercise used the DMPPT to advocate for the establishment of national VMMC programs in collaboration with USAID and UNAIDS [24]. The collaboration included the Office of the U.S. Global AIDS Coordinator (OGAC), the U.S. Centers for Disease Control and Prevention (CDC), the United States Department of Defense (DoD), the World Health Organization, and the Ministries of Health (MOH) from the 13 prioritized African countries.

The DMPPT is a two-partExcel-based modeling tool that estimates HIV infections averted and the cost and net savings associated with VMMC scale-up. A complete description of the DMPPT can be found elsewhere [24]. The second part of the DMPPT, not discussed in detail here, contains a separate costing workbook that calculates cost estrimates, which in turn feed into the DMPPT. In brief, the DMPPT is a compartmental deterministic model, populated with country-specific demographic and epidemiologic estimates from national Spectrum/AIM files. Spectrum is a suite of easy-to-use analytical tools to support health policy decision-making processes. The AIDS Impact Model (AIM) component of Spectrum, used by UNAIDS to generate the national and regional estimates it releases every year, projects the consequences of the HIV epidemic, including the number of people living with HIV, new HIV infections, and AIDS deaths by age and sex. For each scale-up scenario specified, the DMPPT generates a baseline projection of HIV incidence, in which male circumcision coverage is held constant at baseline levels, and a scale-up projection, in which male circumcision is scaled up to a specified level. The term ‘VMMC’ refers only to nationally-implemented medical programs for HIV prevention while the term ‘male circumcision’ includes both traditional and medical male circumcision VMMC is estimated to have an efficacy of 60% [24]. Based on these two separate projections and the country-specific cost information calculated in the second part of the tool, the DMPPT is then able to calculate the projected number of HIV infections averted across the entire population, the cost per HIV infection averted, and subsequent treatment costs averted by provider mix and speed of scale-up.

From 2009 to 2011, DMPPT modeling helped demonstrate the global implications of scaling up male circumcision to 80% coverage by 2015 among males age 15–49 years in the 13 VMMC priority countries. The results showed that to reach 80% coverage would require performing 20.34 million VMMCs between 2011 and 2015, and an additional 8.42 million between 2016 and 2025 to maintain coverage levels [24]. This scale-up was projected to avert 430,000 HIV infections between 2011 and 2015, and almost 3.36 million through 2025 [24]. Furthermore, while the scale-up itself was projected to cost a total of US$2 billion between 2011 and 2025, the projected HIV treatment costs averted meant there would be a net savings of US$16.51 billion. When considered all together, these DMPPT modeling results made a compelling case for significant investment in rapid scale-up of VMMC in these 13 countries.

These findings were shared, and their implications discussed with stakeholders in the 13 priority countries before a special collection in PLOS Medicine published in November 2011 [26]. To support widespread results dissemination, the modelers presented the findings at public, international fora attended by country governments, international donors, and implementing partners. The published results directly influenced the launch of the UNAIDS-WHO Joint Strategic Action Framework (JSAF) to accelerate the scale-up of VMMC in December 2011 [27]. This framework represented a joint effort by key global and national stakeholders, including national Ministries of Health, to ensure that priority VMMC countries developed and implemented national VMMC scale-up strategies.

On World AIDS Day 2011, President Obama challenged the President’s Emergency Plan for AIDS Relief (PEPFAR) to support 4.7 million VMMCs in less than two years, and Secretary of State Clinton announced that PEPFAR would focus on antiretroviral treatment, prevention of mother-to-child transmission, and VMMC to achieve an AIDS-free generation [28–29]. As such, PEPFAR significantly expanded its five-year strategy to include increased investments in support of VMMC in the 13 priority countries [30]. Having effectively advocated for high-level policy changes, the modeling team now needed to tailor their work to the new policy context by producing national and regional estimates of impact and cost-effectiveness.

These global shifts in attention towards VMMC as a priority HIV prevention intervention had significant impact but did not mark the end of the VMMC modeling story. As discussed above, a truly thorough modeling exercise considers the local context within which it is undertaken, and this consideration is a key enabler in translating modeling to policy. Although the 2011 round of DMPPT modeling used country-specific data in the tool, the exercise served primarily for global advocacy purposes. Consultation with in-country teams was necessarily limited, and the modeling was performed remotely rather than in-country. As country-level implementation of VMMC programs proceeded, and with new resources being made available, new questions arose that needed answers. For example, although the initial priority population for VMMC was males age 15–49 years, some countries’ VMMC programs attracted significant numbers of 10-14-year-olds and experienced low demand among males over age 25 years. A second phase of VMMC modeling was initiated that allowed further analyses of impact and cost-effectiveness by age group and sub-national region. The ultimate objective was to facilitate program planning that was truly country-specific.

### DMPPT 2: VMMC modeling for country-specific program planning

As countries expanded their national VMMC programs, implementers reported variations in supply and demand for services by client age group and geographic location. To address this challenge and assist with clarifying and aligning program priorities, modelers under the USAID-supported Health Policy Project created the DMPPT version 2 in 2013. This enhanced model enabled analysis of VMMC impact and cost-effectiveness by age group and sub-national region [31].

The modelers worked closely with country stakeholders to implement the DMPPT 2 in nine countries. The first round of country applications occurred in five countries: Malawi, South Africa, Swaziland, Tanzania, and Uganda, with the second round undertaken in Kenya, Namibia, Lesotho, and Mozambique. In each country, model inputs and analyses were customized based on country stakeholder inputs led by the MOH. Collaborating closely together throughout the process, the modelers and the country teams ensured that the modeling results were directly applicable to countries’ policy decisions. Each country’s results reflected actual experience and were validated by, and relevant to, local stakeholders. This early and continued collaboration, as well as thorough consideration of local contextual factors, are enablers to translating modeling evidence to policy. As a result, DMPPT 2 outputs have been incorporated into program planning for all nine countries [32] and have informed global guidance from PEPFAR and UNAIDS [33].

The national VMMC program in ESwatini (formerly Swaziland) is an illustrative example. After developing a national VMMC policy in 2008, Eswatini’s Ministry of Health aimed to explore the implications of focusing service delivery on specific age groups, based on the heterogeneous demand for services the country had experienced [16]. The results of the application of the DMPPT 2 in Eswatini in 2013 helped the MOH incorporate new evidence to underpin geographic and age prioritization into its Male Circumcision Strategic and Operational Plan for HIV Prevention, 2014–2018 [34,35]. The modeling exercise explored six different scenarios for scale-up based on varying levels of target coverage by client age group. In an effort to balance cost, cost-effectiveness, impact, programmatic feasibility, and consistency with the Extended National Multisectoral HIV and AIDS Framework 2014–2018, the MOH chose a scenario that scaled up to 50% coverage among neonates, 80% coverage among males age 10–29 years, and 55% coverage among males age 30–34 years [35].

In Malawi, use of the DMPPT 2 results also demonstrates direct translation of modeling results to policy. First, an analysis looking at health zones across the country showed that intensifying scale-up would be most cost-effective in two of five zones, namely, the South Western and South Eastern zones [36]. When country stakeholders noted that these did not include Lilongwe—an urban centre with a high HIV prevalence and a strong VMMC program—they suggested an analysis focused on urban versus rural areas (see Table 3 in [36]). This customized analysis showed that scale-up in urban areas in Malawi is three times more cost-effective than in rural areas. Accordingly, the country’s new VMMC strategy prioritizes scale-up in Lilongwe in addition to the South Western and South Eastern zones. Specific coverage targets generated by the DMPPT 2 model have been incorporated into the Voluntary Medical Male Circumcision Strategy and National Operations Plan for Scale Up 2015–2020 [34].

Based on country examples such as these, PEPFAR identified as a key priority for immediacy and magnitude of impact [34], the scale-up of VMMC services to achieve at least 80 percent VMMC coverage in men ages 15–29 (“age-pivot”) [37]. However, when the programme scale-up phase eventually reaches its targets, results from the DMPPT 2 could help support the argument that broadening this age group to include early infant male circumcision (EIMC) and younger age boys is critical for national VMMC programs services during the program sustainability phase.

The successful translation of modeling insights into policy in these examples was facilitated by the following three steps in each country. First, the modelers sought to *identify and engage with a wide array of stakeholders* involved in the country’s VMMC program. Depending on the country, this ranged from 10 to 20 people. Based on guidance from the MOH and the PEPFAR team in each country, various national and global stakeholders were included, with central roles being played by representatives from the MOH, in-country US government agencies, implementing partners, WHO, and UNAIDS. Including anyone who could potentially be involved in future data use ensured early buy-in. Second, the modelers held in-person meetings with stakeholders to *reach consensus on key aspects of the analysis*: the proposed research questions, sources for model inputs, and—importantly—critical assumptions. Gaining consensus on these aspects prior to the analysis allowed for widespread acceptance and ownership of the results at later stages. Furthermore, by shedding light on these inputs, the modelers lessened the “black box” effect that can accompany many modeling exercises. After completing each analysis, a third and final step was to *convene a results dissemination meeting with critical stakeholders*. Together, the modelers and relevant in-country players reviewed, discussed, and agreed on the results and their policy implications, facilitating the process of knowledge translation into policy at country level.

Not only did the DMPPT 2 become recognised as a useful tool, the process highlighted above secured sufficient buy-in from country policymakers for application of the findings. Once stakeholders understood and appreciated the tool and its results, the modeling efforts entered a new phase. The tool was placed directly into stakeholders’ hands along with support to enhance their capacity to use it independently.

### DMPPT 2 online: User-friendly VMMC modeling for program monitoring

Although the main purpose of the DMPPT 2 was to assess the impact and cost-effectiveness of offering circumcision services to different age groups, many countries wanted to use it to monitor program achievements and track their progress toward the revised goals they had set. In its Microsoft Excel format, the DMPPT 2 remained a complex tool that required technical support for its manipulation. Importantly, it was not user-friendly and was difficult to use as a monitoring tool. In response, the DMPPT 2 Online (http://dmppt2.org/) was developed in 2016 and launched in January 2017. A detailed user guide can be found on the tool website. The DMPPT 2 Online is coded in Delphi with outputs provided to the user in a downloadable Excel workbook—allowing for simple, direct transfer into strategic planning documents. These outputs can be visualized graphically and exported for illustrative presentations [38]. For full description of the model, see [31].

The DMPPT 2 Online is a web-based tool that allows PEPFAR country teams and government counterparts to quickly run scale-up scenarios and generate VMMC targets, coverage estimates, and impact projections. Furthermore, given recent emphasis on generating more granular data, the DMPPT 2 Online disaggregates estimates to subnational unit (SNU) level—in most cases, this is the district level. As opposed to the DMPPT 2, the online version can be easily manipulated, making the tool well-suited for program monitoring and future scale-up projections by policymakers. The ability to track progress closely is critical for countries to move from scaling up to maintaining high VMMC coverage levels. The simplicity of the DMPPT 2 Online lends itself to easy data manipulation by end users, as country teams become more familiar with the tool.

When a user accesses the DMPPT 2 Online with personalized and unique login credentials, he or she chooses a scale-up scenario by indicating: (1) a target coverage level for each five-year age group (e.g. 80%), and (2) a target year by which the country should reach the specified coverage. The user can then download the corresponding ‘output table’ (an Excel workbook) for that scenario, which contains a package of useful data for each SNU (Box 1). The first tab in the ‘output table’ is the user-specified scenario or coverage target that will be achieved by the target year.

**Box 1. List of results included in the DMPPT 2 online ‘output table’**

1. **Targets**: The number of VMMCs required in each age group and year to reach the specified coverage target.
2. **Coverage:** The estimated VMMC coverage by age group for the next five years.
3. **Uptake rate:** Expressed as a percentage, the uptake rate is calculated by dividing the number of circumcisions conducted in each age group and year by the number of uncircumcised men in that age group and year; essentially, it measures the reach of the VMMC program by age and year, given the potential client pool (uncircumcised men).
4. **Impact—HIV infections averted:** The projected HIV infections averted over the next 15 years, disaggregated by those attributable to circumcisions conducted to date and those attributable to future circumcisions.
5. **Impact—VMMC per HIV infection averted:** The number of VMMCs required to avert one HIV infection over the next 15 years, based on the user-specified coverage target.
6. **Increase in coverage:** The reach of a program or uptake of service in a given age group in addition to the natural aging from one age band to the next.
7. **DataPack inputs:** Inputs required for PEPFAR country operational planning—Male population and the number of currently circumcised men, age 15–29 years, in the current year. The Datapack is one of the PEPFAR target setting tools for use by countries in making their funding request
8. **Unmet need—uncircumcised male population by age group.** The proportion of the male population that is uncircumcised, by age group.
9. **HIV prevalence:** Proportion of the population with HIV.

Additionally, the user can choose from a list of 11 visualizations to display the results (see Box 2). These visualizations can be downloaded as images for presentation purposes. (Please refer to S1 Annex for a sample of each visualization—provided for Manica province of Mozambique).

**Box 2. List of result visualizations displayed in the DMPPT 2 online**

**Fig 1:**
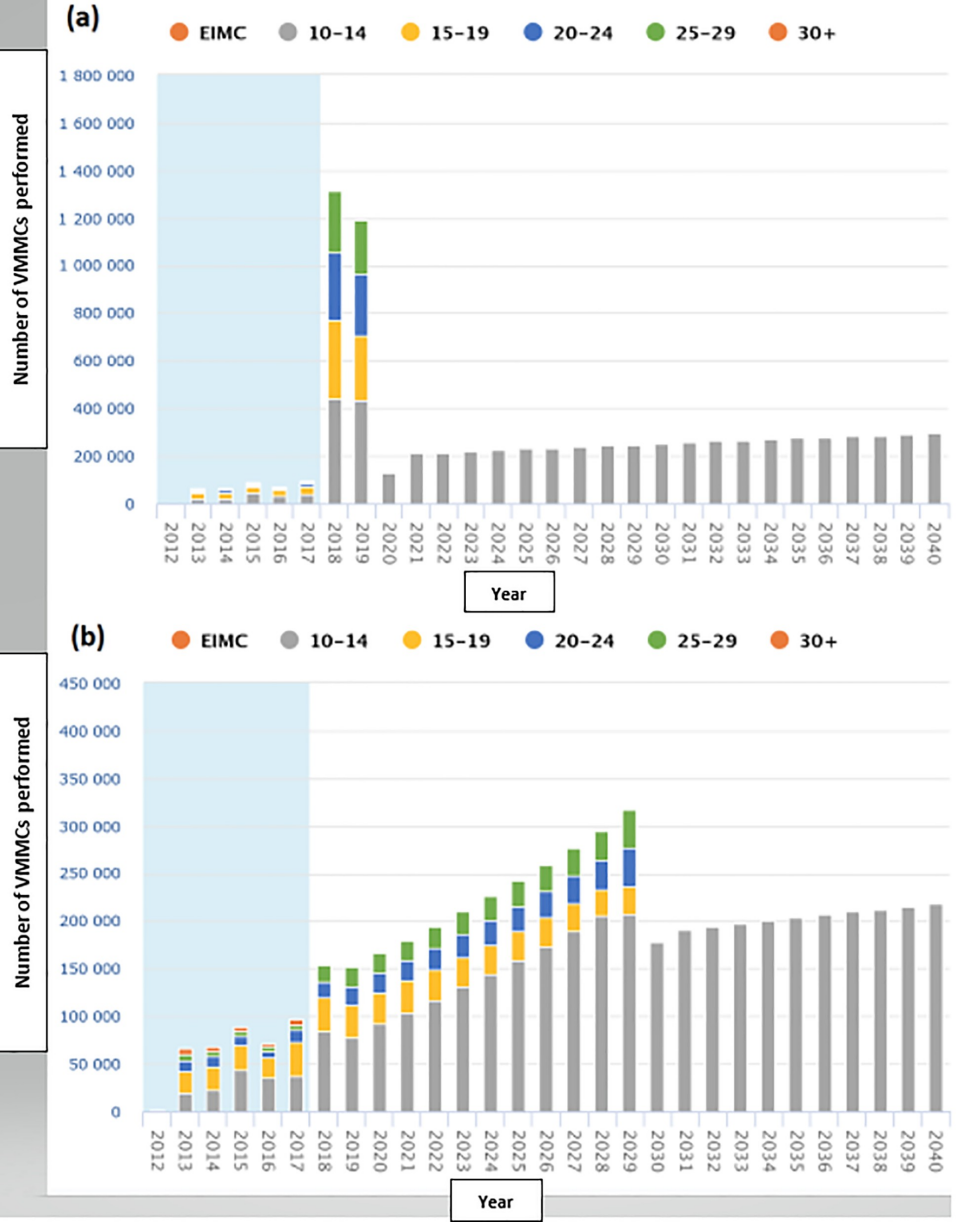
**Result visualization #1 for hypothetical scale-up scenarios:** Number of VMMCs required for: (a) scale-up to 80% coverage among males age 10–29 years by 2020, (b) scale-up to 60% coverage among males age 10–29 years by 2030.

1. **Targets vs. prior achievements:** The number of VMMCs required by age and year to reach and maintain the user-specified coverage level by the target year. This bar graph allows the user to see whether the annual number of projected VMMCs is similar to what has been done in the past or would require many more or fewer circumcisions annually than past achievements. It is a quick visual check for the feasibility of a given set of targets for each SNU. (Fig 1)
2. **Progress in coverage, by age group:** Baseline male circumcision prevalence prior to the VMMC program by age group and SNU compared (in bar graph form) to modeled VMMC coverage estimates at the beginning of a user-specified year.
3. **Progress in coverage, by SNU:** Male circumcision prevalence by SNU before the start of the VMMC program compared (in a bar graph form) to modeled estimates by a user-specified year, in a user-specified age group.
4. **Coverage table by age/SNU:** Modeled estimates of MC coverage (%) by age group and SNU, for a user-specified year. This table uses color-coding to create a ‘heat map.’
5. **Impact—HIV infections averted:** HIV infections averted by SNU, counted over a 15-year period starting in the year following the current one, and displayed in a bar graph.
6. **Efficiency**—**VMMC per HIV infection averted:** The number of VMMCs required to avert one HIV infection by SNU, over a 15-year period starting in the year following the current one. This metric, displayed in a bar graph, takes into account only the future VMMCs needed to achieve and maintain the user-specified coverage target. Efficiency is defined in this context as the number of VMMCs performed per HIV infection averted over 15 years [29].
7. Progress in coverage, by country*: Cross-country comparison (comparable to Result 3): Male circumcision prevalence before the start of the VMMC program compared (in a bar graph form) to modeled estimates by a user-specified year, in a user-specified age group.
8. Coverage table by age/country*: Cross-country comparison (comparable to Result 4): Modeled estimates of MC coverage by age group, for a user-specified year. This table uses color-coding to create a ‘heat map.’
9. **Age prioritization analysis**
  - **Age prioritization analysis 1, VMMCs performed by age group and year:** The number of VMMCs already conducted by the national program in each age group and year, displayed in a bar graph.
  - **Age prioritization analysis 2, Uptake of VMMC services by age group and year:** The uptake rate is the number of circumcisions in a given age group in a given year divided by the number of uncircumcised men in that age group and year (represented as a percentage, in a bar graph).
  - **Age prioritization analysis 3, Increase in VMMC coverage:** The increase in VMMC coverage, by age group, from one year to the next, displayed as a bar graph.
10. Uptake of VMMC services by country: Age prioritization analysis*: Cross-country comparison showing the uptake rate or VMMC services, represented as a percentage, in a bar graph. Results 7, 8 and 10 are only available when the user checks the “Show global outputs” box in the “Options” menu on the left side of the screen
11. **Unmet need: uncircumcised male population by age group.** The proportion of the male population that is uncircumcised by age group, represented as a percentage in a pyramid.

*.

To illustrate the use of the result visualizations, consider Fig 1 generated from the DMPPT 2 Online, which displays two hypothetical versions of result visualization #1 (targets vs. prior achievements) in panels (a) and (b). In Fig 1A, the user had chosen to scale up to 80% coverage by 2020 among males age 10–29 years. However, Fig 1A shows the number of VMMCs required in 2018 and 2019 was significantly higher than the historical achievements of the program (bars in the blue-shaded region on the left), possibly indicating an unrealistic target. When the user changed the scenario to *60% coverage* among males age 10–29 years by *2030* (Fig 1B), the targets looked much more realistic given the history of the program and indicate a smoother scale-up than in Fig 1A.

The range of results in the DMPPT 2 Online—both in the output tables and in the various visualizations—allows any user to create a tailored analysis and pull out the relevant outputs on their own, thus building capacity for using the model for program monitoring. DMPPT 2 Online developers will continue to build on this local capacity by facilitating regular trainings and updates, as well as collating all relevant training materials in an easy-to-access online training hub. Thus, the team has established a plan for sustained use of modeling for policy and program planning.

## Case study lessons learned

In summary, the three modeling tools (DMPPT, DMPPT 2, and the DMPPT 2 Online) demonstrate a range of uses for modeling in HIV programming—from advocacy to program monitoring. The DMPPT helped to quantify the benefits of VMMC scale-up, providing the push needed for a global commitment from donors and country governments. Countries that implemented the DMPPT 2 then made strategic, evidence-informed decisions on their national targets and VMMC policies. Ongoing use of the DMPPT 2 Online is now helping these countries track the progress and impact of their programs, down to the district level.

Researchers sometimes conceptualize modeling exercises without fully appreciating the nature of policy development, how best it can be informed by modeling, and the importance of active stakeholder engagement throughout the process. Working with the DMPPT, DMPPT 2, and DMPPT 2 Online, modelers and country teams collaborated together to ensure that model results were validated by and relevant to the country stakeholders, giving due consideration to the policymaking process at each stage. In fact, each of the tools was created in response to needs that had been specifically articulated by policymakers and program planners.

When modeling studies do not go through the process highlighted above, the resultant use of results may be low [39,40]. In the concluding section, we summarize a comprehensive framework that can be successfully applied when translating modelling results to policy and programming.

## Conclusions and framework for success

In reflecting on the VMMC modeling case study, we have highlighted enablers to the effective use of modeling data to inform decision-making, namely, responding to articulated policy needs, collaborating with stakeholders, considering local context, enumerating assumptions, and spending the necessary time to conduct a thorough analysis.

Building on these enablers and lessons learned through the VMMC case study, we outline here a ‘Framework for Success’ for using mathematical modeling to inform health policy, based on the application of DMPPT to VMMC (Box 3). Though not entirely new, compiling this potential framework for success could serve as a guide for future modeling efforts to ensure that results are not purely theoretical but rather produce co-owned, concrete programmatic decisions relevant to the end-users they serve.

**Box 3. ‘Framework for success’ in applying mathematical modeling to inform health policy using VMMC as a case study**

1. Identify a champion (or champions)
  - To gain traction with country stakeholders, a local champion or influencer promoting the value of using modeling evidence for decision-making sets the stage for a joint process.
2. Engage stakeholders early and often
  - Engaging *the most relevant* stakeholders early and often helps ensure that the research question and anticipated results are appropriate and useful for policymaking.
3. Encourage consensus
  - Agreeing on data inputs, assumptions, and limitations ahead of time removes the “black box” effect of modeling—where the inner workings are a mystery and the results have no context.
4. Customize analyses
  - Ensuring that analyses are customized to the specific local context of a country (via stakeholder input) improves the analyses and inspires confidence that the outputs are relevant for local policymaking.
5. Build capacity
  - Developing a user-friendly tool thorough stakeholder engagement and validation helps build local capacity and facilitates communication, enhancing understanding of results and their implications.
6. Establish a plan for sustainability
  - By facilitating regular trainings and updates, modeling teams can help ensure that tools are fully integrated in the policymaking process and continue to be relevant to decision makers.

Each step in this ‘Framework for Success’ has been crucial to converting results from the VMMC modeling analyses into evidence-informed policies. When applying these steps to future modeling applications, it is essential to keep in mind the time and resources that go into the process. While this kind of commitment can become costly, it ensures that the results are relevant to efficient use of resources. Through intensive engagement and careful attention to each step, mathematical modeling can inform decisions aimed at improving existing health policy—ultimately resulting in better health outcomes.

## Supporting information

S1 Annex Full set of DMPPT 2 online result visualizations for Manica province, Mozambique. (DOCX)

## Abbreviations

AIDS: acquired immune deficiency syndrome
ART: antiretroviral therapy
CDC: Centers for Disease Control and Prevention
DMPPT: Decision Makers’ Program Planning Tool
DoD: United States Department of Defense
JSAF: UNAIDS-WHO Joint Strategic Action Framework
HIV: human immunodeficiency virus
MOH: Ministry of Health
OGAC: Office of the U.S. Global AIDS Coordinator
PEPFAR: President’s Emergency Plan for AIDS Relief
SNU: subnational unit
UNAIDS: Joint United Nations Programme on HIV/AIDS
USAID: United States Agency for International Development
VMMC: voluntary medical male circumcision
WHO: World Health Organization
