# Supplementary material for: Using mathematical modeling to inform health policy: A case study from voluntary medical male circumcision scale-up in eastern and southern Africa and proposed framework for success
Source: PLoS One. 2019 Mar 18;14(3):e0213605. doi: 10.1371/journal.pone.0213605 (PMC6422273; doi:10.1371/journal.pone.0213605)

**S1 Annex.** Full set of DMPPT 2 Online result visualizations for Manica Province, Mozambique.

For all below results, the user-specified target scenario is to reach 80% coverage among males 10-29 by the beginning of 2020. For more details on the DMPPT 2 Online, please access it at the following site: <http://avenirhealth.org/policytools/DMPPT2/>

1. **Targets vs. prior achievements:** The number of VMMCs required by age and year to reach and maintain the user-specified coverage level by the user-specified target year. The purpose of this bar graph is for the user to see whether the annual number of target VMMCs is similar to what has been done in the past (bars shaded in blue), or would require many more or fewer circumcisions annually than past achievements. It is a quick visual check for the feasibility of a given set of targets for each SNU.


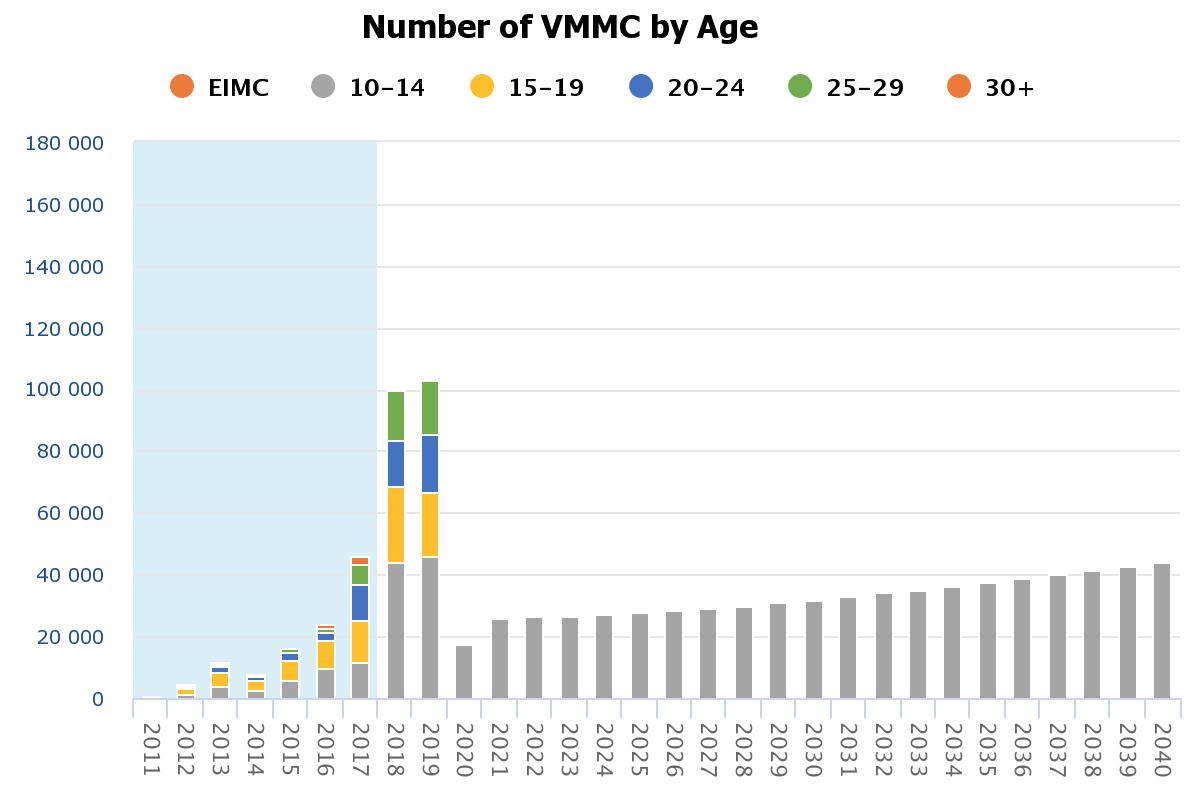


1. **Progress in coverage, by age group:** Male circumcision prevalence (%) by age group and SNU before the start of the VMMC program compared to modelled VMMC coverage estimates at the beginning of a user-specified year.


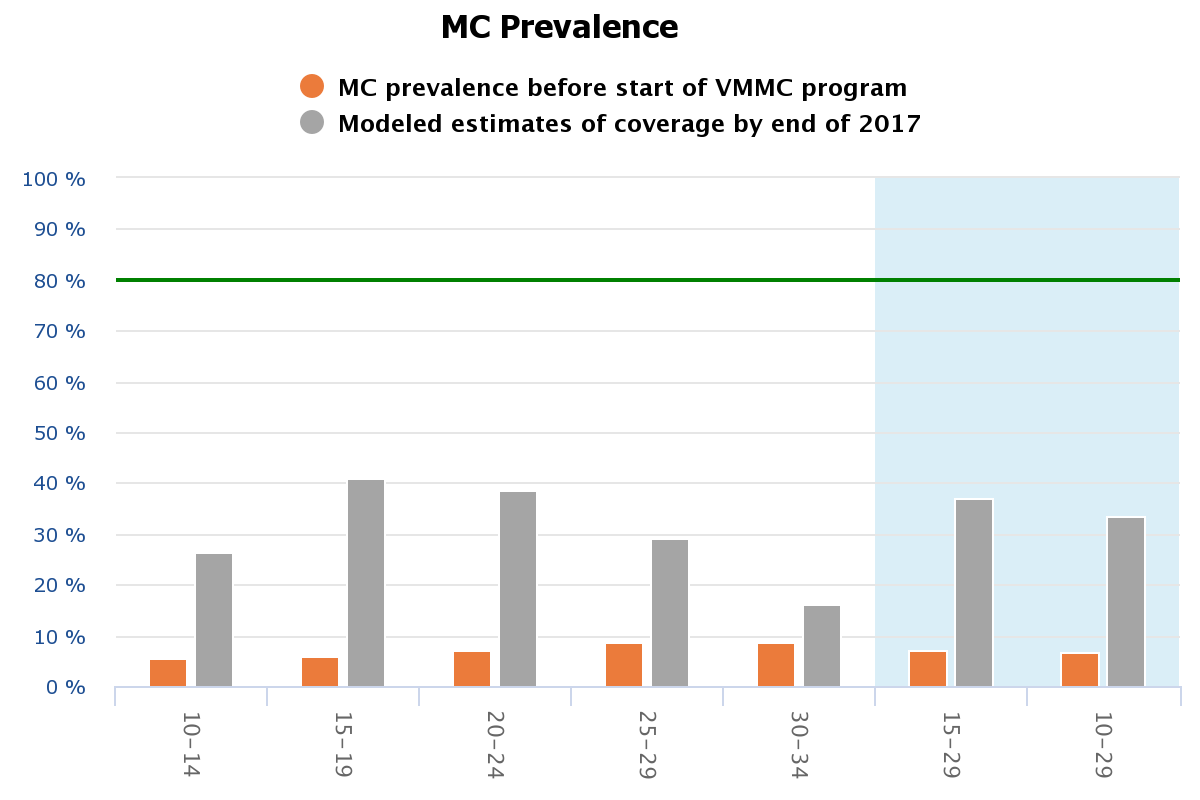


1. **Progress in coverage, by SNU:** Male circumcision prevalence (%) by SNU before the start of the VMMC program compared to modelled estimates by a user-specified year, in a user-specified age group.


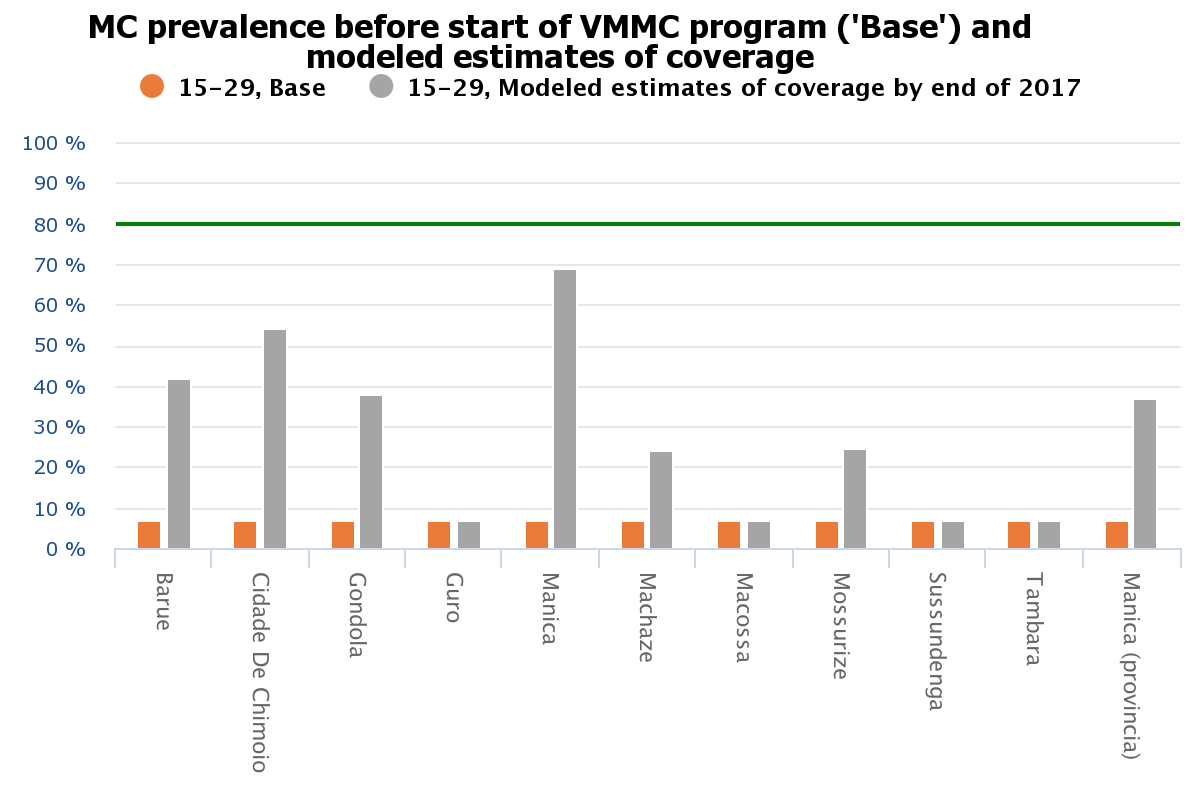


1. **Coverage table by age/SNU:** Modelled estimates of MC coverage (%) by age group and SNU, for a user-specified year (here, end of 2017). This table uses color-coding to create a ‘heat map’ effect. Color-coding: Green is above 70%, yellow is between 50% and 70% and red is below 50%.


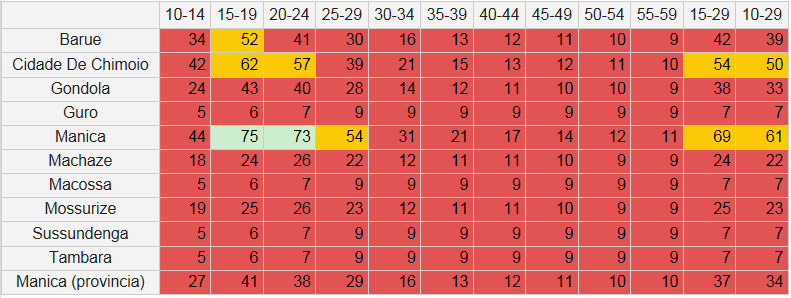


1. **HIV infections averted:** HIV infections averted by SNU, counted over a fifteen-year period starting in 2017, and displayed in a bar graph. 'Program VMMCs to date' refers to historical VMMCs already conducted by the national program. 'Scale-up VMMCs only' refers to the future VMMCs needed to achieve and maintain the user-specified coverage target.


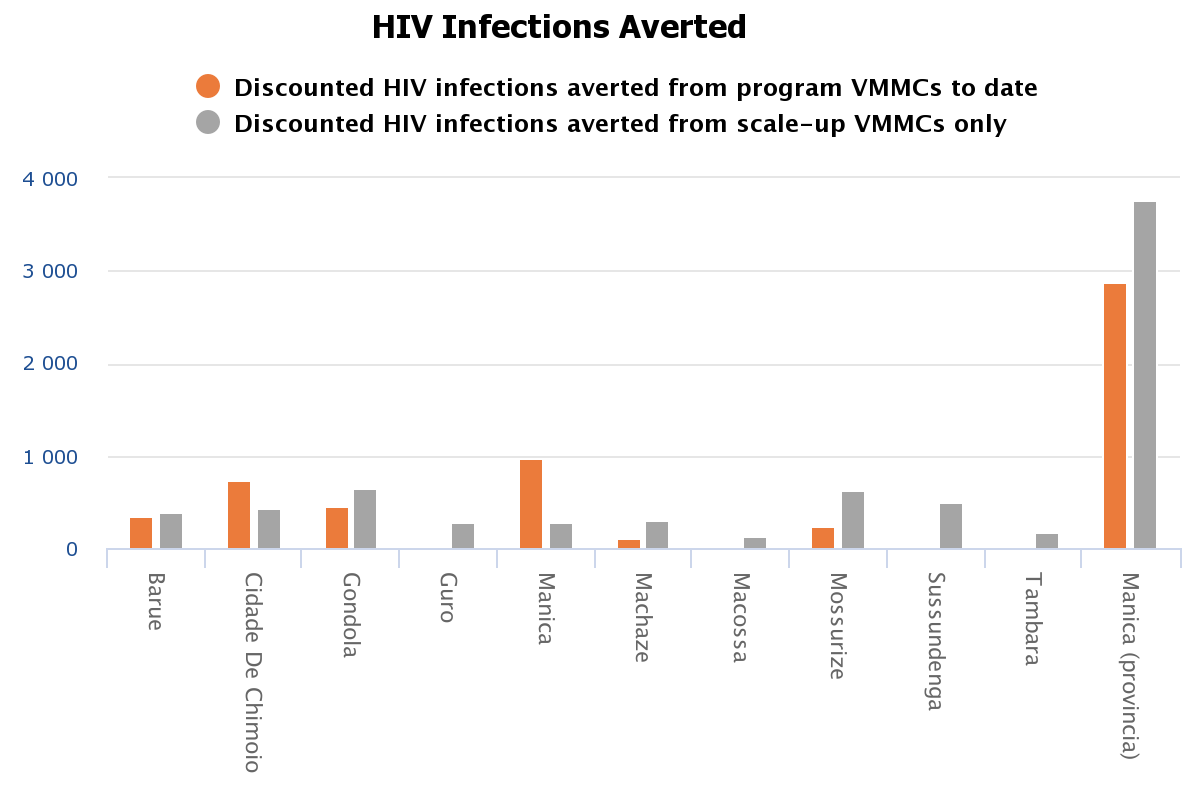


1. **VMMC per HIV infection averted:** The number of VMMCs required to avert one HIV infection by SNU, over a fifteen-year period starting in 2017. This metric, displayed in a bar graph, only takes into account the 'scale-up VMMCs' only, i.e., the future VMMCs needed to achieve and maintain the user-specified coverage target.


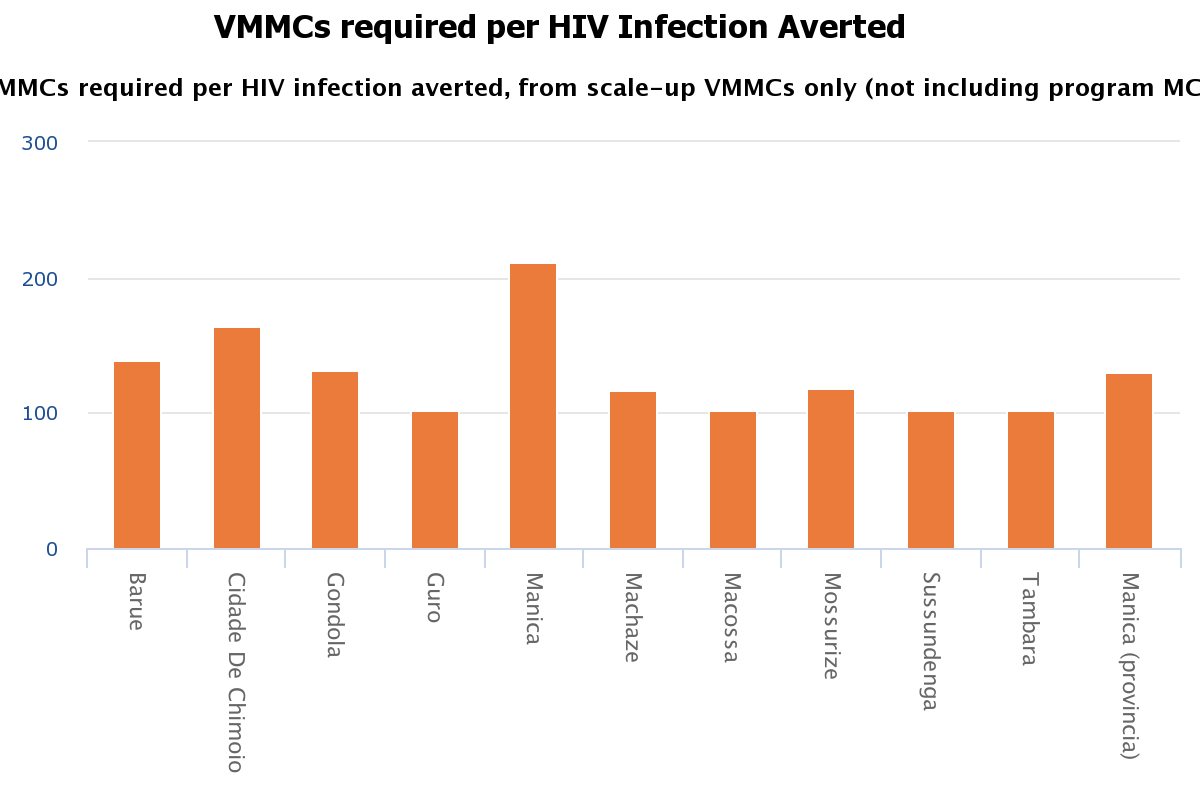


1. **Progress in coverage, by country:** Cross-country comparison (comparable to Result 3): Male circumcision prevalence (%) before the start of the VMMC program compared to modelled estimates by a user-specified year, in a user-specified age group.


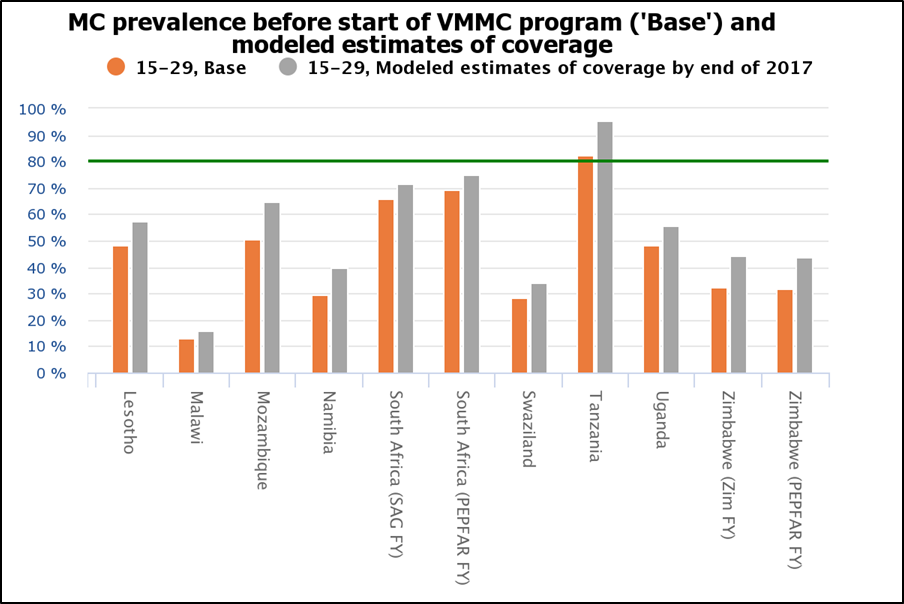


1. **Coverage table by age/country:** Cross-country comparison (comparable to Result 4): Modelled estimates of MC coverage (%) by age group, for a user-specified year (here, end of 2017). This table uses color-coding to create a ‘heat map’ effect. Green is above 70%, yellow is between 50% and 70% and red is below 50%.


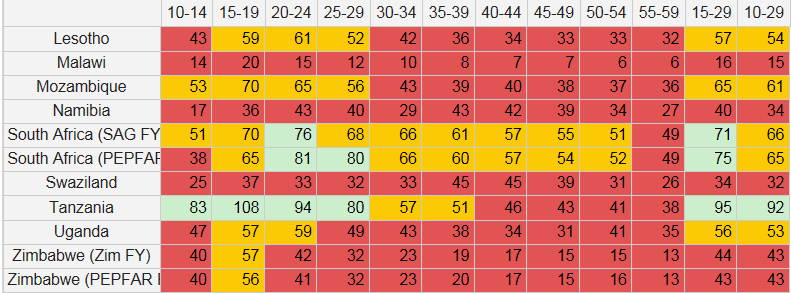


1. **Age pivot analysis 1, VMMCs done by age group and year:** The number of VMMCs already conducted by the national program in each age group and year.


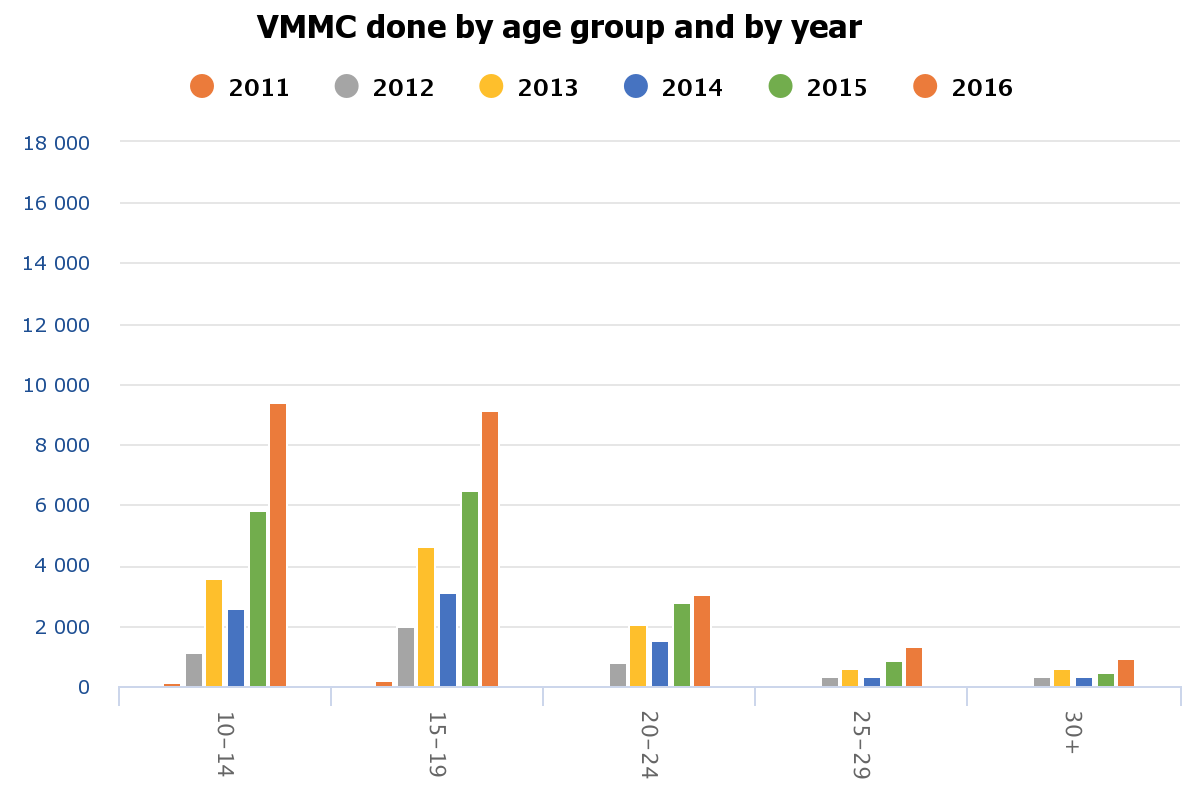


1. **Age pivot analysis 2, Uptake of VMMC services by age group and year:** The uptake rate is the number of circumcisions in a given age group in a given year divided by the number of uncircumcised men in that age group and year (represented as a percentage).


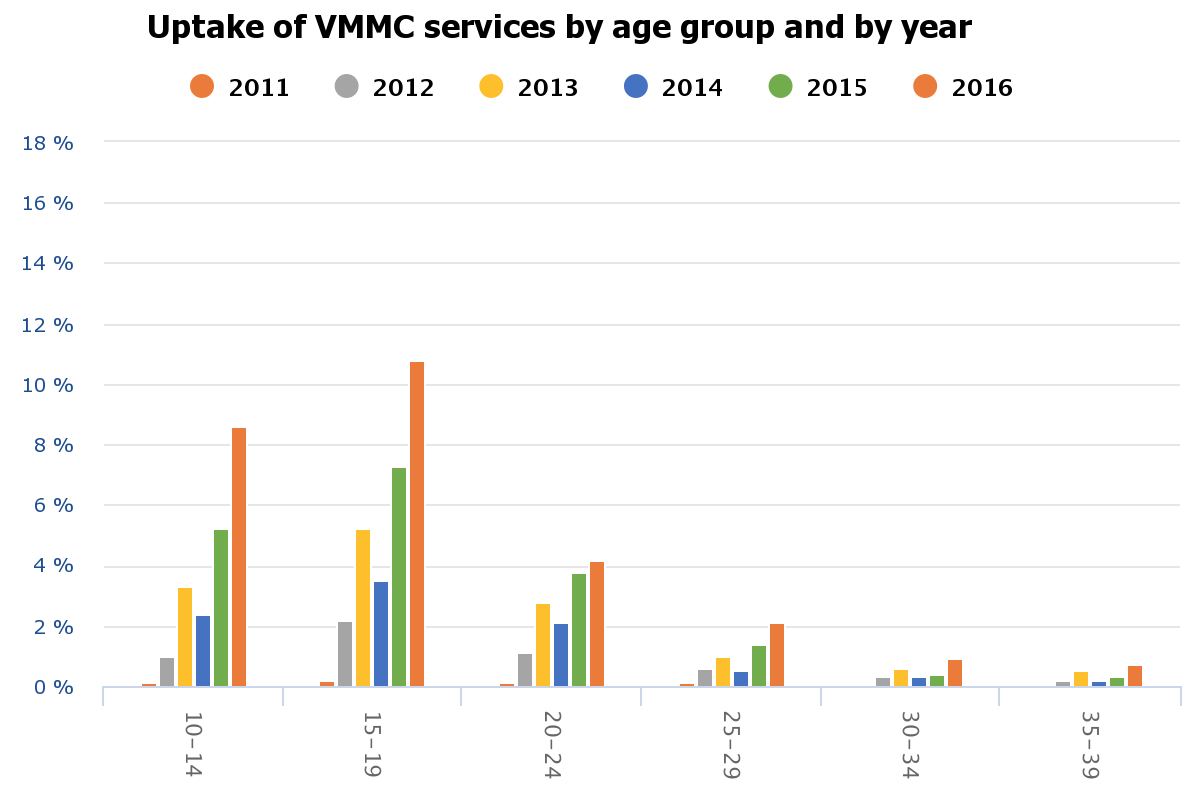

Supplement: S1 Annex — (DOCX) [file pone.0213605.s001.docx]
